# Four-factor Prothrombin Complex Concentrate for Reversal of Factor Xa Inhibitors versus Warfarin in Life-threatening Bleeding

**DOI:** 10.5811/westjem.2020.11.47931

**Published:** 2021-02-26

**Authors:** Megan A. Rech, Dalila Masic, Drayton A. Hammond

**Affiliations:** * Loyola University Medical Center, Department of Pharmacy, Maywood, Illinois; † Stritch School of Medicine, Loyola University Chicago, Department of Emergency Medicine, Maywood, Illinois; ‡ Rush University Medical Center, Department of Pharmacy, Chicago, Illinois; § Rush Medical College, Department of Internal Medicine, Chicago, Illinois

## Abstract

**Introduction:**

Factor Xa (fXa) inhibitor reversal for life-threatening bleeding is controversial due to a lack of high-quality evidence. The purpose of this study was to determine the hemostatic efficacy of four-factor prothrombin complex concentrate (4F-PCC) for the reversal of fXa inhibitors compared to warfarin for life-threatening bleeding.

**Methods:**

This was a multicenter, retrospective cohort study at two academic medical centers between January 1, 2014–December 31, 2019, which included patients who presented to the emergency department with a life-threatening bleed necessitating anticoagulation reversal with 4F-PCC. The primary endpoint was achievement of hemostatic efficacy after 4F-PCC administration.

**Results:**

Of the 525 patients who had an order for 4F-PCC during the study period, 148 patients met the criteria for inclusion (n = 48 fXa inhibitor group; n = 100 warfarin group). Apixaban (52.1%) and rivaroxaban (45.8%) were the most commonly used fXa inhibitors. Effective hemostasis was similar between groups (79.2% fXa inhibitor group vs 85% warfarin group, p = 0.38). This was consistent across all types of bleeding. Thrombotic events were rare in both groups (2% vs 3%).

**Conclusion:**

This multicenter, retrospective cohort study demonstrated that using 4F-PCC for treatment of life-threatening bleeding produced effective hemostasis in patients on fXa inhibitors and warfarin.

## INTRODUCTION

Factor Xa (fXa) inhibitors (eg, apixaban and rivaroxaban) are a class of direct oral anticoagulants that are widely used for a variety of indications, including venous thromboembolism and atrial fibrillation.^1^,^2^ Use of these agents has steadily increased over the last decade. This is in part because of their ease of use compared to warfarin, which requires frequent laboratory monitoring and dietary modifications and which interacts with numerous medications due to metabolism by a number cytochrome P450 enzymes, posing safety risks. Compared to warfarin, fXa inhibitors appear to have a lower rate of intracerebral hemorrhage, with annual rates of 0.1–0.2% compared to 0.3–0.6% of patients on warfarin.^3^,^4^ However, whereas four-factor prothrombin complex concentrate (4F-PCC) and vitamin K are generally considered the standard of care for reversal of life-threatening bleeding secondary to warfarin (due to warfarin’s availability it has been more extensively studied), reversal of fXa inhibitors in this setting remains controversial due to a lack of high-quality evidence.^5^–^8^

Several national and international guidelines endorse the use of 4F-PCC for the reversal of fXa inhibitors; however, its exact place in therapy overall and in relation to andexanet alfa is discordant among these guidelines.^1^,^2^,^9^–^11^ Although andexanet alfa was specifically designed for reversal of fXa inhibitors, it has not seen widespread use because many institutions have not approved it due to a lack of robust evidence, including a comparator group in the available studies, questionable risk of thromboembolism, and the poor value proposition and cost-effectiveness of the therapy.^12^,^13^ The range of recommendations for its use include the following: 4F-PCC as a first-line therapy for fXa inhibitor reversal^1^,^10^,^11^; 4F-PCC as a first-line therapy as an alternative to discontinuation of fXa inhibitors alone (eg, meaning that perhaps no reversal agent would be appropriate)^2^; and 4F-PCC as a second-line agent after andexanet alfa.^9^ These differences stem from the relatively poor quality of evidence for both agents and include a degree of expert opinions. The limited data available are comprised of small, single-center studies that lack a comparator group.^14^–^20^ The purpose of this study was to determine the hemostatic efficacy of 4F-PCC for the reversal of fXa inhibitor-related, life-threatening bleeding compared to 4F-PCC for warfarin-related life-threatening bleeding.

## METHODS

This was a multicenter, retrospective cohort study conducted at two urban, academic medical centers between January 1, 2014–December 31, 2019. The study included patients who presented to the emergency department (ED) with a life-threatening bleed necessitating anticoagulation reversal with 4F-PCC. To be included in the study, there had to be confirmation of warfarin or fXa inhibitor use (ie, apixaban, betrixaban, edoxaban, or rivaroxaban) prior to presentation, which necessitated rapid reversal for life-threatening bleeding. Patients were excluded if any of the following criteria were present: age less than 18 years; receipt of 4F-PCC outside of the ED setting or at an outside hospital prior to arrival; receipt of 4F-PCC for any indication aside from life-threatening bleeding; concurrent factor VII use; history of heparin-induced thrombocytopenia; or known disseminated intravascular coagulation. Both institutions’ institutional review boards approved the research protocol.

Life-threatening bleeding was treated according to institutional protocols at the discretion of the treating services. Both institutions preferentially used 4F-PCC for the reversal of life-threatening bleeding in patients on fXa inhibitors or warfarin during the study period. Institutional protocols at both sites recommended dosing of 4F-PCC at 50 factor IX units per kilogram for fXa inhibitor reversal and between 25–50 IX units/kg for warfarin reversal based on a pre-treatment international normalized ratio (INR) value. The primary endpoint was achievement of hemostatic efficacy after 4F-PCC administration as defined by the Scientific and Standardization Subcommittee on Control of Anticoagulation of the International Society of Thrombosis and Hemostasis Scientific (ISTH) for the assessment of the effectiveness of major bleeding management.^21^

**Population Health Research Capsule**

What do we already know about this issue?

*Factor Xa (fXa) inhibitors are used for venous thromboembolism and atrial fibrillation. Evidence regarding their reversal in the setting of life-threatening bleeding is limited*.

What was the research question?

What was the hemostatic efficacy of four-factor prothrombin complex concentrate (4F-PCC) for the reversal of fXa inhibitor-related life-threatening bleeding compared to 4F-PCC for warfarin-related life-threatening bleeding?

What was the major finding of the study?

*Effective hemostasis was similar between groups and was consistent across all types of bleeding; thrombotic events were rare in both groups*.

How does this improve population health?

*Using 4F-PCC for treatment of life-threatening bleeding produced effective hemostasis in patients on both fXa inhibitors and warfarin*.

Hemostasis for intracranial hemorrhage was defined as stabilization at or less than a 35% increase in hematoma volume on imaging. All patients had repeat imaging based on treatment protocols. Hemostasis for visible bleeding was defined as cessation of visible bleeding within four hours of 4F-PCC administration. Hemostasis for non-visible bleeding was defined as stable hemoglobin at 48 hours post-4F-PCC administration. Hemostasis was assessed by one of the study investigators upon data collection. Secondary endpoints were the number of transfusions of packed red blood cells, platelet, and fresh frozen plasma, discharge disposition, intensive care unit length of stay, hospital length of stay, and hospital and 30-day mortality. Safety endpoints were any adverse event during hospitalization (ie, deep vein thrombosis [DVT], pulmonary embolism, ischemic stroke, arterial thrombus, myocardial infarction, hypersensitivity reaction, transfusion-related acute lung injury, and transfusion-associated circulatory overload).

### Statistical Analyses

Baseline and clinical characteristics were characterized using descriptive statistics. We assessed normality of continuous variables using the Shapiro-Wilk test. Normally distributed continuous variables were analyzed using Student’s t-test. We used the Mann-Whitney U test to analyze on-parametric data. A chi-square or Fisher’s exact test was used to compare categorical variables, as appropriate. We analyzed data using STATA version 15 (StataCorp, College Station, TX).

## RESULTS

**Figure 1:**
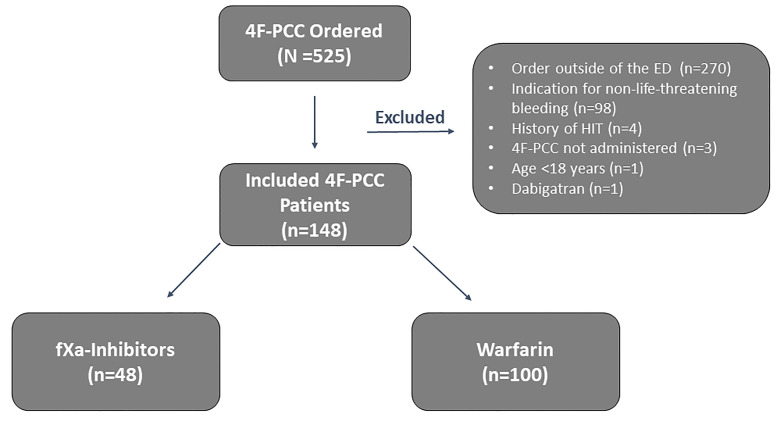
Study diagram. *4F-PCC*, four-factor prothrombin complex concentrate; *ED*, emergency department; *HIT*, heparin-induced thrombocytopenia; *fXa*, factor Xa.

Of the 525 patients who had an order for 4F-PCC during the study period, 148 patients met the criteria for inclusion (n = 48 fXa inhibitor group; n = 100 warfarin group; Figure 1). The most common reasons for exclusion were receipt of 4F-PCC outside of the ED (n = 270) and use for non-life-threatening bleeding (n = 98).

Baseline demographics were similar between groups (Table 1). Patients in the fXa inhibitor group were older (78.4 years fXa inhibitor group vs 73.9 years warfarin group, *P* = 0.03), while patients in the warfarin group had a higher incidence of end-stage renal disease (2.1% vs 18%, *P* < 0.01). Apixaban (52.1%) and rivaroxaban (45.8%) were the most commonly used fXa inhibitors, with only one patient (2.1%) on edoxaban. Most patients in both groups were on an oral anticoagulant for atrial fibrillation-associated stroke prevention. Anticoagulation for mechanical mitral valve only occurred in the warfarin group (0% vs 19%, *P* < 0.01). Concomitant antiplatelet use was similar between groups, with aspirin being the most common agent (29.7% vs 26%, *P* = 0.69), followed by clopidogrel (16.7% vs 21%, *P* = 0.53). Only two patients, both in the warfarin group, were on dual antiplatelet therapy.

The most common indication for 4F-PCC in both groups was intracranial bleeding, which occurred more frequently in the warfarin group (52.1% vs 67%, *P* = 0.02; Table 2). Visible bleeding was more common in the fXa inhibitor group (31.3% vs 15%, *P* = 0.02), while non-visible bleeding was similar between groups (16.7% vs 17%). Four-factor prothombin complex concentrate was administered more often during weekdays and day shifts in both groups. Baseline laboratory parameters were similar between groups, although warfarin patients had higher INR (1.2 vs 3.2, *P* < 0.01). Patients in the fXa-group received a higher total and weight-based dose of 4F-PCC than the warfarin group. Most doses in both groups were deemed appropriate according to institutional guidelines. There was little difference between groups in time to 4F-PCC initiation (106.5 minutes vs 140 minutes, *P* = 0.12).

The primary endpoint of effective hemostasis was similar between groups (79.2% vs 85%, *P* = 0.38; Table 3). This was consistent across all types of bleeding with no differences observed in intracranial bleeding, visible bleeding, or non-visible bleeding hemostasis. No patients received additional hemostatic agents or coagulation factors at 48 hours after 4F-PCC. All efficacy and safety secondary endpoints were similar between groups. Only three adverse effects occurred overall. One patient in each group developed DVT and one ischemic stroke occurred in the warfarin group.

## DISCUSSION

This multicenter, retrospective cohort study demonstrated that using 4F-PCC for treatment of life-threatening bleeding produced effective hemostasis in patients on both fXa inhibitors and warfarin. Hemostasis was high overall, occurring in 79.2% of the fXa inhibitor group and 85% of the warfarin group. Hemostasis rates were consistent with previously reported literature.^8^,^15^,^19^,^20^ The addition of a comparator group (eg, warfarin) in our study allowed for a frame of reference to be available, unlike prior studies that analyzed 4F-PCC use in fXa inhibitor-induced bleeding. This is important as 4F-PCC is generally considered the treatment of choice for warfarin-related bleeding, but guideline recommendations are more heterogeneous when it comes to recommending 4F-PCC for fXa-inhibitor-related bleeding due to a paucity of evidence guiding treatment decisions.^1^,^2^,^9^,^11^

Previous studies of fXa inhibitor-related bleeding reversal have observed similar efficacy rates as this study (range: 72.4–85%), although most describe single-center efforts with low patient enrollment rates and no comparator group.^8^,^15^,^19^,^20^ The largest retrospective cohort study to date included 663 patients with intracranial hemorrhage, of whom 433 were evaluated for hemostatic efficacy.^8^ Within this patient cohort, efficacy was deemed excellent or good (according to hematoma expansion ≤20% or 20.1–35%, respectively) in 81.8% of patients. A prospective observational study of 66 patients receiving a fixed dose of 2000 IX units did a post hoc analysis for effective hemostatic according to ISTH criteria and found 68% of patients achieved effectiveness.^15^ A meta-analysis including 10 case series of 340 patients found that only two studies used the ISTH criteria to define hemostasis.^22^ In these studies, the effective management of major bleeding was achieved in 69% of patients (95% confidence interval [CI], 61–76%). There was a low rate of thromboembolic events within 30 days (3% [95% CI, 0–6%]). None of the included studies had a comparator arm. Our study enhances the current literature with the addition of a comparator group, which provides a frame of reference for clinicians to consider when determining oral anticoagulant choice and potential outcomes if a life-threatening bleed occurs.

If a reversal agent predisposes patients to developing a thrombotic event following use, its utility may be greatly diminished. Thus, careful monitoring for adverse effects is important. In this study, safety outcomes occurred very infrequently and were similar between groups. One patient experienced a DVT in the fXa inhibitor group, compared to one DVT and one stroke in the warfarin group. The fXa inhibitor patient required subsequent anticoagulation and did not experience any further adverse effects or mortality. Other studies have reported similar adverse effects.^8^,^15^,^19^,^20^ A single-center, retrospective cohort study of 4F-PCC used for either reversal of fXa inhibitor induced life-threatening bleeding or need for emergent procedure also found only one adverse effect, a DVT.^14^ A large, multicenter study found thrombotic events in 3.8% of patients.^8^ Thus, according to this study and previous literature, it appears that 4F-PCC is a relatively safe intervention in the treatment of fXa inhibitor- and warfarin-related bleeding.

## LIMITATIONS

This study has several limitations that warrant consideration. First, despite being one of the largest studies of fXa inhibitor-induced bleeding reversed with 4F-PCC, it is a retrospective cohort study of a relatively limited number of patients, with only 48 patients in the fXa inhibitor group. We attempted to improve upon previous literature by including two academic centers and comparing 4F-PCC efficacy and safety to warfarin, where it has been established as the standard of care for reversal in the setting of life-threatening bleeding.^1^,^2^,^6^,^9^,^11^ However, this may have introduced bias as fXa inhibitors likely cause less severe bleeding than warfarin.^23^ Second, in an attempt to have the most complete data possible in terms of timing and documentation, we excluded the 270 patients experiencing life-threatening bleeding outside of the ED, which may have limited our external validity. Additionally, a previous study found that time to intervention could potentially affect outcomes.^24^ While we collected data from ED arrival to administration of 4F-PCC, the time of last dose of anticoagulant was not readily available. Finally, thrombotic adverse effects could have occurred after discharge and may have been missed due to a relatively short follow-up period, especially if patients reported to an outside hospital that was not connected with our electronic health record.

## CONCLUSION

This multicenter, retrospective cohort study demonstrated that using 4F-PCC for treatment of life-threatening bleeding produced effective hemostasis in patients on fXa inhibitors and warfarin. Although larger, prospective comparative studies are needed to determine the efficacy of 4F-PCC as a reversal agent for fXa inhibitor-related, life-threatening bleeding, this study adds to the existing literature supporting use of 4F-PCC for this indication based on the hemostatic efficacy and safety of this intervention.

## Figures and Tables

**Table 1:** Baseline characteristics of patients treated for life-threatening bleeding.

Characteristic	fXa-Inhibitors (n=48)	Warfarin (n=100)	P-value
Male gender, n (%)	28 (52.1)	64 (64)	0.17
Age at bleed (years), median (IQR)	78.4 (68.9 – 83.9)	73.9 (62.6 – 82.3)	0.03
BMI (kg/m2), median (IQR)	26.8 (24 – 31.8)	28.6 (28.6 – 32.3)	0.24
Race, n (%)			0.67
Black	9 (18.8)	20 (20)	
White	31 (64.6)	55 (55)	0.11
Hispanic	3 (6.3)	9 (9)	0.11
Other	5 (10.4)	16 (16)	<0.01
Comorbidities			0.19
Atrial fibrillation	40 (83.3)	70 (70)	0.53
Cancer	13 (27.1)	16 (16)	0.24
End-stage renal disease	1 (2.1)	18 (18)	0.34
Heart failure	13 (27.1)	38 (38)	--
Venous thromboembolism	8 (16.7)	21 (21)	
SOFA score on admission, median (IQR)	3 (2 – 5)	2 (1 – 5)	
GCS score on admission, median (IQR)	14 (10 – 15)	15 (11 – 15)	
Anticoagulation, n (%)			
Warfarin	--	100 (100)	
Rivaroxaban	22 (45.8)	--	
Apixaban	25 (52.1)	--	
Edoxaban	1 (2.1)	--	
Indication for anticoagulation, n (%)			
Atrial fibrillation	39 (81.3)	68 (68)	0.12
Cancer-related venous thromboembolism	1 (2.1)	1 (1)	0.59
Deep venous thrombosis	8 (16.7)	10 (10)	0.25
History of venous thromboembolism	2 (4.2)	7 (7)	0.50
Mechanical mitral valve	0 (0)	19 (19)	<0.01
Pulmonary embolism	4 (8.3)	8 (8)	0.95
Ventricular assist device	0 (0)	4 (4)	0.16
Concomitant antiplatelet, n (%)			
Aspirin	11 (29.7)	26 (26)	0.69
Clopidogrel	8 (16.7)	21 (21)	0.53
Prasugrel	2 (4.2)	1 (1)	0.20
Dual antiplatelet therapy	0 (0)	2 (2)	0.33
Baseline laboratory parameters, median (IQR)			
INR	1.2 (1.1 – 1.9)	3.2 (2.4 – 6.3)	<0.01
Hemoglobin, g/dL	11.3 (8.2 – 13.2)	11.5 (9.8 – 13.2)	0.53
Platelets, ×10^9^/L	219 (161 – 257)	212 (172 – 257)	0.82

*fXa*, factor Xa; *IQR*, interquartile range; *kg*, kilogram; *m2*, meter squared; *BMI*, body mass index; *SOFA*, sequential organ failure assessment; *GCS*, Glasgow Coma Scale; *INR*, international normalized ratio; *g*, gram; *dL*, deciliter; *L*, liter.

**Table 2:** Anticoagulation reversal characteristics.

Characteristic	fXa-Inhibitors (n=48)	Warfarin (n=100)	P*-*value
Type of Bleed, n (%)			0.02
Intracranial bleeding	25 (52.1)	67 (67)	
Visible bleeding	15 (31.3)	15 (15)	
Non-visible bleeding	8 (16.7)	17 (17)	
4F-PCC day of the week, n (%)			0.65
Weekday (Monday – Friday)	38 (79.2)	75 (75)	
Weekend (Saturday, Sunday)	10 (20.8)	25 (25)	
4F-PCC shift, n (%)			0.65
Day (0701 to 1900)	38 (79.2)	76 (76)	
Evening (1901 to 0700)	10 (20.8)	24 (24)	
Laboratory parameters, median (IQR)			
INR after 4F-PCC	1.2 (1.1 – 1.3)	1.2 (1.1 – 1.4)	0.65
Hemoglobin, g/dL, 48 hours	9.9 (8.9 – 12)	10.2 (8.7 – 12.2)	0.93
Platelets, ×10^9^/L, 48 hours	185 (141 – 226)	186 (144 – 216)	0.68
4F-PCC dose, units, median (IQR)	3932 (3212 – 4516)	2265 (1740 – 3136)	<0.01
4F-PCC dose, units/kg, median (IQR)	49.9 (47.3 – 52.4)	27.5 (24.4 – 35.3)	<0.01
Time to 4F-PCC, minutes, median (IQR)	106.5 (64 – 216)	140 (77 – 240)	0.12
Appropriate 4F-PCC dose, n (%)	43 (89.6)	84 (84)	0.35

*fXa*, factor Xa; *4F-PCC*, four-factor prothrombin complex concentrate; *IQR*, interquartile range; *g*, gram; *dL*, deciliter; *L*, liter; *INR*, international normalized ratio.

**Table 3:** Hemostatic efficacy.

	fXa-Inhibitors (n=48)	Warfarin (n=100)	P*-*value
Primary endpoint			
Effective hemostasis, n (%)	38 (79.2)	85 (85)	0.38
Hemostasis by type of bleed			
Intracranial bleeding hemostasis, n (%)	n = 25	n = 25	
Hematoma volume stable or increased by <35% compared to baseline	19 (76)	59 (86.8)	0.21
Deterioration in GCS at 24 hours	2 (8)	6 (8.8)	0.90
Need for further hemostatic agents or coagulation factors at 48 hours	4 (16)	25 (36.8)	0.06
Visible bleeding hemostasis, n (%)	n = 15	n = 15	
Cessation of visible bleeding within 4 hours of 4F-PCC administration	14 (93.3)	15 (100)	0.29
Need for further hemostatic agents or coagulation factors at 48 hours	12 (80)	12 (80)	>0.99
Non-visible bleeding hemostasis, n (%)	n = 8	n = 17	
Stable hemoglobin at 24 hours after 4F-PCC	8 (100)	17 (100)	>0.99
Need for further hemostatic agents or coagulation factors at 48 hours	4 (50)	12 (75)	0.32
Secondary outcomes			
Mortality, n (%)			
Hospital	8 (16.7)	14 (14)	0.69
30-day	9 (18.8)	17 (17)	0.81
Length of stay, median (IQR)			
Intensive care unit	2 (1 – 7)	3 (2 – 7)	0.31
Hospital	6 (4 – 10)	7 (4 – 13)	
Adverse event during hospitalization, n (%)	1 (2.1)	2 (2)	0.97
Packed red blood cells within 24 hours of 4F-PCC, median (IQR)	17 (35.4)	24 (24)	0.15
Platelet transfusions within 24 hours of 4F-PCC, median (IQR)	4 (8.3)	21 (21)	0.05
Fresh frozen plasma within 24 hours of 4F-PCC, median (IQR)	6 (12.5)	23 (23)	0.13

*fXa*, factor Xa; *GCS*, Glasgow Coma Scale; *4F-PCC*, four-factor prothrombin complex concentrate; *IQR*, interquartile range.
